# Fibroblast growth factor 23, endothelium biomarkers and acute kidney injury in critically-ill patients

**DOI:** 10.1186/s12967-019-1875-6

**Published:** 2019-04-11

**Authors:** Fernanda Macedo de Oliveira Neves, Camila Barbosa Araújo, Daniele Ferreira de Freitas, Bianca Fernandes Távora Arruda, Leonardo José Monteiro de Macêdo Filho, Vivian Brito Salles, Gdayllon Cavalcante Meneses, Alice Maria Costa Martins, Alexandre Braga Libório

**Affiliations:** 1 Medical Sciences Postgraduate Program, Department of Clinical Medicine, Universidade Federal do Ceará, Avenida Abolição, 4043 Ap 1203, Fortaleza, Ceará CEP 60165-082 Brazil; 2 Medical Sciences Postgraduate Program, Universidade de Fortaleza–UNIFOR, Fortaleza, Ceara Brazil; 3 Medical Course, Universidade de Fortaleza–UNIFOR, Fortaleza, Ceará Brazil; 4 Department of Clinical and Toxicological Analysis, Faculty of Pharmacy, Federal University of Ceara, Fortaleza, Ceara Brazil

**Keywords:** Acute kidney injury, Endothelium, Fibroblast growth factor 23, Mediation, ICU

## Abstract

**Background:**

Fibroblast growth factor 23 (FGF23) and endothelium-related biomarkers have been related to AKI in critically-ill patients. Also, FGF23 is associated with endothelial dysfunction. In this study, we investigated if elevated FGF23 association with severe AKI is mediated by several endothelial/glycocalyx-related biomarkers.

**Methods:**

Prospective cohort study with critically-ill patients. Blood samples were collected within the first 24 h after ICU admission. Severe AKI (defined according to KDIGO stage 2/3) was the analyzed outcome.

**Results:**

265 patients were enrolled and 82 (30.9%) developed severe AKI—defined according to KDIGO stage 2/3. Blood samples to biomarkers measurement were collected within the first 24 h after ICU admission. After adjustment for several variables, FGF23, vascular cell adhesion protein 1 (VCAM-1), angiopoietin 2 (AGPT2), syndecan-1 and intercellular adhesion molecule-1 (ICAM-1) were associated with severe AKI. The individual indirect effects of VCAM-1, AGPT2 and syndecan-1 explained 23%, 31%, and 32% of the total observed effect of FGF23 on severe AKI, respectively. ICAM-1 showed no statistically significant mediation. When all three endothelium-related biomarkers were included in a directed acyclic graph (DAG), the Bayesian network learning suggested the following causal association pathway FGF-23 → syndecan-1 → VCAM-1 → AGPT2 → severe AKI.

**Conclusions:**

The association between FGF23 and AKI are mediated by endothelium-related biomarkers, mainly VCAM-1, AGPT2 and syndecan-1. Moreover, the statistical models show that syndecan-1, a biomarker of endothelial glycocalyx dysfunction, seems to be the initial mediator between FGF23 and severe AKI.

**Electronic supplementary material:**

The online version of this article (10.1186/s12967-019-1875-6) contains supplementary material, which is available to authorized users.

## Background

Acute kidney injury (AKI) is a common complication of critical illness and is associated with markedly increased hospital length of stay, mortality, and cost [1–3]. Disordered mineral metabolism is a common complication of chronic kidney disease (CKD) [4], but only recently it has been studied in AKI [5–9]. Decreased 1,25-dihydroxyvitamin D (1,25-vitD) levels and hypocalcemia associated with reduced or elevated parathyroid hormone (PTH) levels have been reported in small samples of patients with established AKI [6, 10–12]. Also, fibroblast growth factor (FGF) 23, a potent phosphaturic hormone released by osteocytes, play an important role in phosphate and vitamin D homeostasis and it is elevated in AKI [6–8].

In the CKD setting, elevated FGF23 levels are now recognized as a key feature of dysregulated mineral metabolism, and are one of the most robust predictors of cardiovascular disease in this patient population [13]. More recently, FGF23 has emerged as a biomarker related to AKI and death in critically-ill patients [6, 7, 9] and following cardiac surgery [8].

While the pathogenesis of AKI in critically-ill patients is complex, recent evidence suggests an important role for microvascular endothelial injury and endothelial glycocalyx dysfunction in its pathogenesis [14, 15]. This is supported by studies suggesting that biomarkers related to endothelial cell activation (such VCAM-1) [16], glycocalyx lesion (syndecan-1) [17, 18] and endothelial growth factor (angiopoietin-2 and VEGF-1) [19–21] are associated with AKI.

It is not known whether FGF23 is simply a disease severity marker or directly contributes to adverse outcomes in critically-ill patients. If a direct role is present, the downstream consequences of FGF23 in critically-ill patients leading to AKI are largely unknown; however, elevated plasma FGF23 levels have been implicated in the pathogenesis of endothelial dysfunction [22–24], immunosuppression [25] and inflammation [26] in CKD patients, making these effects possible FGF23 pathways in AKI.

In this study, we hypothesized that (1) elevated FGF23 levels in critically-ill patients is associated with severe AKI and (2) FGF23 association with severe AKI is mediated, at least in part, by endothelium-related biomarkers. To explore these hypotheses, we aimed to use mediation analysis and directed acyclic graph. Although the cross-sectional design of our study precludes direct proof of the causal mechanisms that we propose, statistical mediation and causal inference provides a framework in which we may formally test the observed data for evidence of such mechanisms. Mediation can show whether some or all of the significance of the association between an exposure and an outcome is statistically explained by the effect of the exposure on the potential mediator. The analysis also gives an estimate of the amount of the observed effect of the exposure on the outcome that can be explained by the action of the exposure through the mediator if the hypothesized mechanism exists.

Because endothelial biomarkers can have unknown and complex interactions, we also used Bayesian networks to infer interrelationship between the biomarkers and severe AKI. Bayesian networks are graphical models that leverage conditional independencies between variables to describe joint multivariate probability distributions [27]. These analyses are better discussed below in “Methods” section. Although this evidence is necessarily circumstantial, it can still provide important support and motivation for more definitive investigations.

## Methods

### Study design

This is a post hoc analysis of a prospective observational study [28] we conducted with 265 patients admitted to ICUs at Hospital Geral de Fortaleza (Ceará, Brazil) between June of 2016 and January of 2018. We collected and stored EDTA plasma aliquots within 24 h of ICU admission. All samples were collected in the morning (from 6:00 to 8:00 a.m.). Samples were stored at − 80 °C within 2 h of collection. All patients provided written informed consent and all protocols were approved by our hospital’s Institutional Review Board.

### Enrollment criteria

Inclusion criteria were age > 18 years and admission to a medical or surgical ICU. Exclusion criteria were: (1) anticipated ICU stay < 24 h; and (2) serum creatinine (sCr) > 4.0 mg/dL, submitted to dialysis or previous renal transplantation. Patients were recruited within the first 24 h of ICU stay and followed up to ICU discharge. The institution’s research ethics board approved the study. Informed consent was obtained from patients or parents/guardians before participation, with assent where appropriate.

### Data collection and study procedures

Demographic data, medical history (comorbidities) and severity parameters were recorded within the first 24 h of ICU stay to calculate the Acute Physiology and Chronic Health Evaluation (APACHE) II score. During the first 7 days of ICU stay, sCr level was measured daily and the patient was evaluated regarding the need for mechanical ventilation, use of vasoactive drugs or exposure to nephrotoxic drugs (amphotericin, vancomycin, aminoglycoside). Baseline renal function was defined as the sCr level on ICU admission.

### Laboratory measurements

FGF23 was measured using a C-terminal ELISA kit (R&D Systems, Minneapolis, MN, USA). The intra-assay coefficient of variation was 6.1%.

Syndecan-1 was measured as a biomarker of endothelial glycocalyx injury (Abcam, Cambridge, MA, USA). The intra-assay coefficient of variation was 6.2%. Intercellular adhesion molecule-1 (ICAM-1), a marker of endothelial cell activation, was measured using a commercially available enzyme-linked immunosorbent assay kit (Life Technologies Brasil, São Paulo, Brazil), with an intra-assay coefficient of 8.4%. Also, vascular cell adhesion protein 1 (VCAM-1) was measured using a commercially available enzyme-linked immunosorbent assay kit (Abcam, Cambridge, MA, USA), with an intra-assay coefficient of 5.9%. Angiopoietin-2 was measured using an enzyme-linked immunosorbent assay (R&D Systems, Minneapolis, MN, USA). The intra-assay coefficient of variation was 5.3% and vascular endothelial growth factor (VEGF), an angiogenic protein known to cause endothelial activation and increase microvascular permeability, was measured using an enzyme-linked immunosorbent assay (R&D Systems, Minneapolis, MN, USA). All measured were performed in duplicate. Serum levels of creatinine, ionized calcium and phosphate were measured for clinical purposes by the hospital laboratory.

Additional analyses were also performed in a nested case–control subcohort of 41 participants with severe AKI and 41 participants with normal renal function at ICU admission (estimated glomerular filtration rate > 90 mL/min/1.73 m [2]) and without developing AKI (no sCr increment greater than 50% of baseline renal function or 0.3 mg/dL). Cases were matched 1:1 to controls based on age, gender and baseline renal function. Plasma levels of intact parathyroid hormone (iPTH), 25-hydroxyvitamin D (25-vitD) and 1,25-vitD were measured in this nested case–control subcohort. iPTH was measured using a chemiluminescent immunoassay (Beckman Coulter, Fullerton, CA), with an intra-assay coefficient of 3.2%. 25-vitD and 1,25-vitD were measured using immunoaffinity enrichment and liquid chromatography–tandem mass spectrometry.

### Outcome

Our outcome was severe AKI (stage 2/3) within the first 7 days of ICU stay according to the criteria established by the Kidney Disease Improving Global Outcomes (KDIGO) Work Group. In summary, doubling of baseline sCr or need for renal replacement therapy. Because we could not exclude some patients were admitted already with AKI, we considered new or worsening AKI taking sCr on ICU admission as baseline.

### Statistical analysis

Normality of data was assessed, and data are reported as mean and standard deviation (SD) or median and IQR (25th–75th percentiles) when appropriate. Baseline characteristics were compared using a 2-sample t test or Mann–Whitney test for continuous variables, whereas dichotomous variables were assessed with χ^2^ test or Fisher exact test. Simple correlations between continuous variables were analyzed using Spearman’s rank correlation coefficient. Non-normal distributions were natural log-transformed for additional analysis. Several multivariable logistic regressions were used to assess the association of FGF 23 and endothelial-related biomarkers with severe AKI. All models were adjusted for age, gender, body mass index (BMI) greater than 30 kg/m [2], Charlson comorbidity index, surgical admission, use of vasoactive drugs, need for mechanical ventilation, exposure to nephrotoxic drugs and APACHE II score at ICU admission.

Mediation analyses were performed when appropriate based on the logistic regression results to assess the hypothesized associations of FGF23 and endothelial-related biomarkers with severe AKI. Specifically, the mediation analysis was performed when (1) the exposure was significantly correlated to the mediator and outcome (after adjustment for confounders) and (2) the mediator was significantly correlated to the outcome. Indirect effects and confidence intervals were estimated by bootstrapping with 5000 resamples using the PROCESS Statistical Package for SPSS (PROCESS version 2—note that version 3 does not run dichotomous outcomes and SPSS, version 20.0, 2011; SPSS Inc., Chicago, IL) [29]. Statistically significant mediation is established when the indirect effect is significantly different from zero, with full mediation defined by the additional attenuation of the association between independent and dependent variables into nonsignificance after inclusion of the mediator variable or variables.

Also, to suggest a pathway between FGF23, endothelial-related biomarkers and severe AKI, we performed a Bayesian network learning analysis. To perform directed acyclic graph (DAG), we permitted a priori any causal pathway between biomarkers and variables. The only restriction was that all significant interdependence with arrow “from” severe AKI was corrected and the direction of the arrow was “to” severe AKI. This was performed to maintain the temporal causal assumption. Secondly, we produced a consensus model by learning 200 Bayesian networks and keeping the arrows that appear at least ≈ 60% of the time (as estimated from the data). The algorithm used was hill climbing with the Bayesian Information Criteria score. In the plot, arrow thickness is in proportion to the frequency reported n order to validate the model learning strategy we perform 10 runs of 10-fold cross-validation and measure the predictive accuracy for severe AKI given all the other variables. We used the package *“bnlearn”* package for R [30]. All comparisons are two-tailed, with *p *< 0.05 considered significant.

## Results

### Patient characteristics

**Fig. 1:**
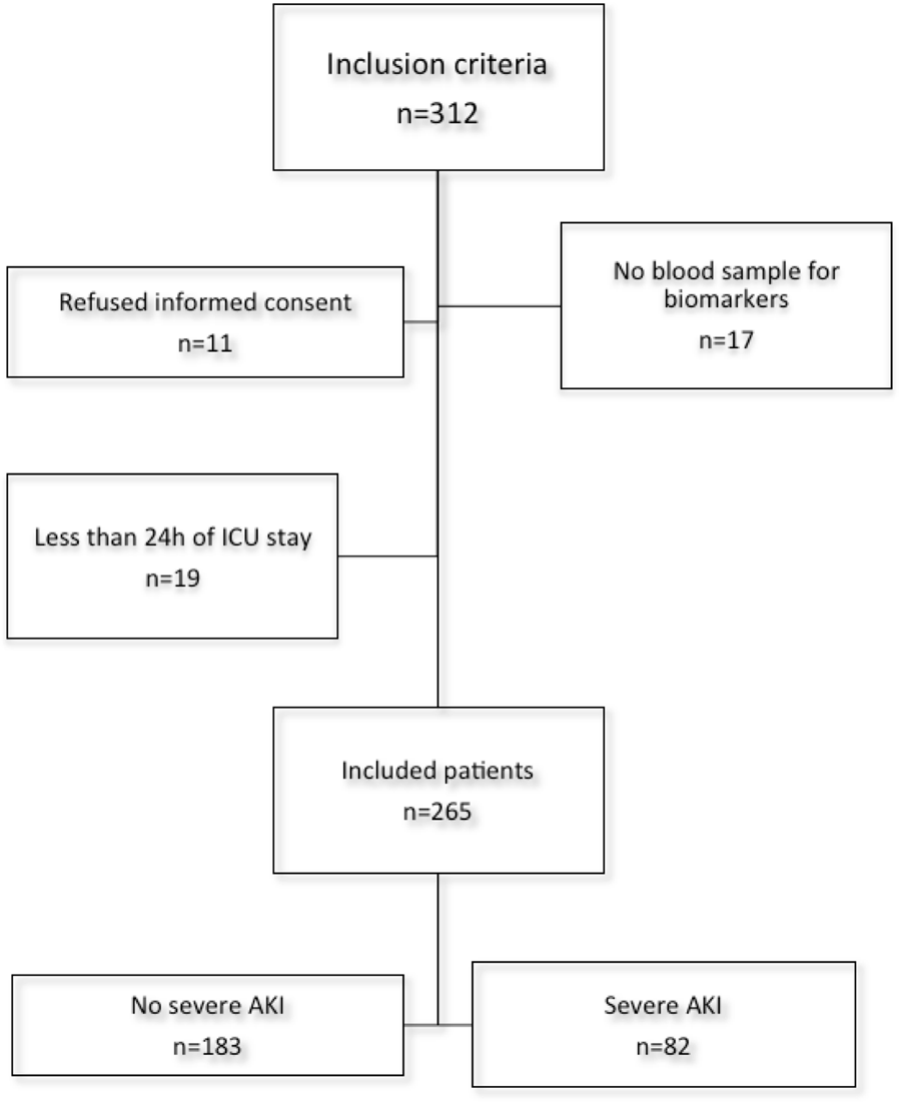
Flowchart of patients and exclusion criteria

Of the 312 patients admitted to the ICU during the study period and considered for inclusion, 47 were excluded due to the following reasons: patient refused to sign the informed consent (*n *= 11), absence of blood sample to measure biomarkers (n = 17), less than 24 h of ICU stay (n = 19)—Fig. 1. We enrolled 265 patients (50.6% males) admitted to the ICU. The mean age was 51.4 ± 17.8 years. One-hundred and nine patients (41.1%) previously had arterial hypertension and 50 (18.9%) had diabetes mellitus. The median (IQR) APACHE II score was 17 (13–22), 160 (60.4%) required mechanical ventilation, and 88 (33.2%) had a surgical admission. Eighty-two patients (30.9%) developed severe AKI. Additional baseline characteristics according to severe AKI status are shown in Table 1.

**Table 1:** Baseline characteristics stratified by AKI status

Characteristic	No-severe AKI (n = 183)	Severe AKI (n = 82)	p
*Demographics*			
Age, mean ± SD	51.2 ± 17.2	51.7 ± 19.1	0.82
Male, *n* (%)	94 (51.4)	40 (48.8)	0.70
BMI greater than 30 kg/m^2^	34 (18.6)	22 (26.8)	0.14
Diabetes mellitus	31 (16.9)	19 (23.1)	0.23
Charlson comorbidity index, median (IQR)	1 (1–2)	1 (1–3)	0.52
*Baseline kidney function*			
Baseline sCr level, median (IQR)	0.6 (0.4–0.7)	0.9 (0.5–1.7)	< 0.001
eGFR, median (IQR)	109 (92–124)	85 (49–115)	< 0.001
Nephrotoxic drugs	28 (15.3)	27 (32.9)	0.002
*Disease severity*			
Sepsis, *n* (%)	48 (26.2)	34 (41.5)	0.015
Surgical ICU	77 (42.1)	11 (13.4)	< 0.001
Mechanical Ventilation, *n (*%)	68 (37.2)	52 (63.4)	< 0.001
Vasoactive drugs (n, %)	49 (26.8)	50 (61.0)	< 0.001
APACHE II score, median (IQR)	15 (11–20)	21 (17–27)	< 0.001
*Plasma FGF23, median (IQR)*	135.3 (10.4–544.3)	927.1 (197.6–1232.6)	< 0.001

*AKI* acute kidney injury, *eGFR* estimated glomerular filtration rate, *sCr* serum creatinine

Age: years; sCr: mg/dL; eGFR: mL/min/1.73 m^2^; FGF23: RU/mL

### Association between FGF23 and endothelium-related biomarkers

Univariate associations of endothelial-related biomarkers, each assessed individually, with FGF 23 are presented in Additional file 1: Table S1. As expected, there was a high interrelationship between endothelial-related biomarkers. FGF23 directly correlated with all endothelial-related biomarkers, except VEGF-1. The strongest association of FGF23 was observed with syndecan-1 (r_s_ = 0.412, p < 0.001).

### Plasma mineral metabolite levels

FGF23 directly correlated with phosphate (*r*_s_ = 0.197, p = 0.002) and, in the nested case–control subcohort, inversely correlated with 1,25-vitD (*r*_s_ = − 0.27, p = 0.01) level. We observed no significant correlation between FGF 23 and 25-vitD (r_s_ = 0.10, p = 0.33) levels.

### FGF 23 and endothelial-related biomarkers are associated with severe AKI

**Fig. 2:**
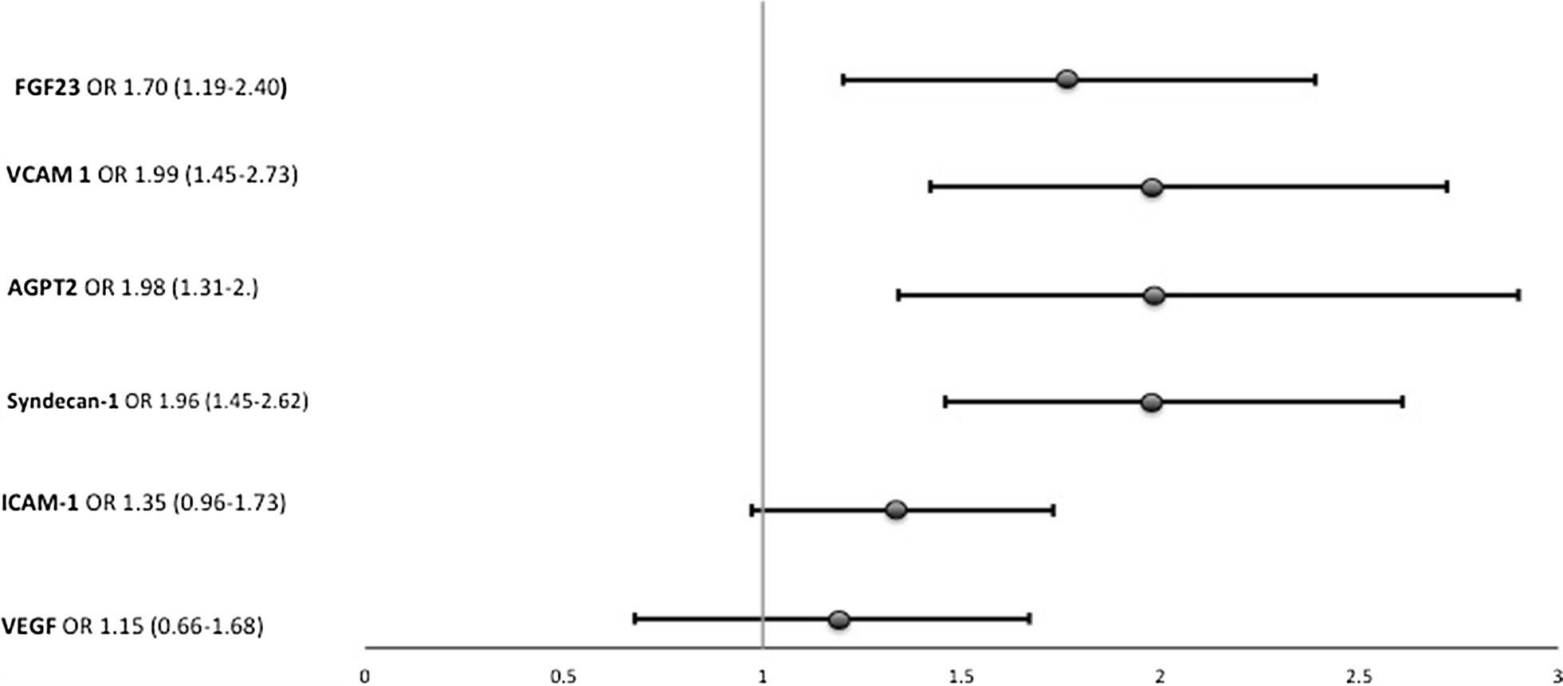
FGF23 and endothelial-related biomarker levels associated with increased risk of AKI/death after adjusted for age, gender, Charlson comorbidity index, BMI greater than 30 kg/m^2^, surgical admission, use of vasoactive drugs, need for mechanical ventilation, use of nephrotoxic drugs and APACHE II score at ICU admission. ORs are shown per 1 unit of SD of log-transformed values for each biomarker

Median plasma endothelial-related biomarker values, according to severe AKI status, are shown in Additional file 2: Table S2. All measured biomarkers, except VEGF, were higher in severe AKI patients. Figure 2 shows adjusted associations of individual biomarkers with severe AKI. FGF23, syndecan-1, VCAM-1 and angiopoietin-2 were strongly associated with severe AKI (p < 0.001). ICAM-1 only showed a statistically significant trend in the association with severe AKI. After adjustments, there was no association between serum phosphate, calcium, iPTH and vitamin D metabolites with severe AKI, as shown in Additional file 3: Table S3).

### Mediation analysis

**Fig. 3:**
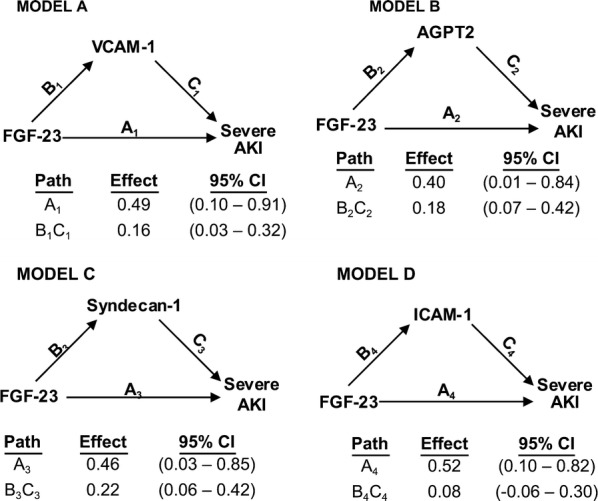
Mediation analyses of the association between FGF23 and severe AKI. Path models and mediation analyses describe mediation of the association between FGF23 and severe AKI through endothelial-related biomarkers individually. Path effects are reported as Odds-ratio scale of natural log-transformed values of biomarkers. Models are adjusted for age, gender, Charlson comorbidity index, BMI greater than 30 kg/m^2^, surgical admission, use of vasoactive drugs, need for mechanical ventilation, use of nephrotoxic drugs and APACHE II score at ICU admission. Residual direct effects are labeled as path A in each model, and indirect effects are labeled as letters B and C

Results of mediation analyses are summarized in Fig. 3. The association between FGF23 and AKI remained significant after including each endothelial-related biomarker individually; excluding some endothelial-related biomarker individually is responsible for total mediation of such association. However, we tested whether the association between FGF 23 and severe AKI was partially mediated by VCAM-1, AGPT2, syndecan-1 and ICAM-1 (models A-D, respectively). Because VEGF and vitamin D metabolites showed no independent association with severe AKI, we did not test whether these biomarkers showed any mediation. ICAM-1 (model D) showed no significant mediation regarding the association between FGF 23 and severe AKI. Regarding the other biomarkers, the individual indirect effects of VCAM-1 (model A), AGPT2 (model B) and syndecan-1 (model C) were, each one of them, significant but not eliminate the significance of the residual direct effect of FGF 23 on severe AKI. The indirect effect in each model explained 24%, 31%, and 33% of the total observed effect, respectively. Because diabetes mellitus can damage the endothelium, we performed a sensitivity analysis including only patients with diabetes mellitus (n = 50). The mediation results using parsimonious models were similar—Additional file 4: Figure S1.

### Directed acyclic graph

**Fig. 4:**
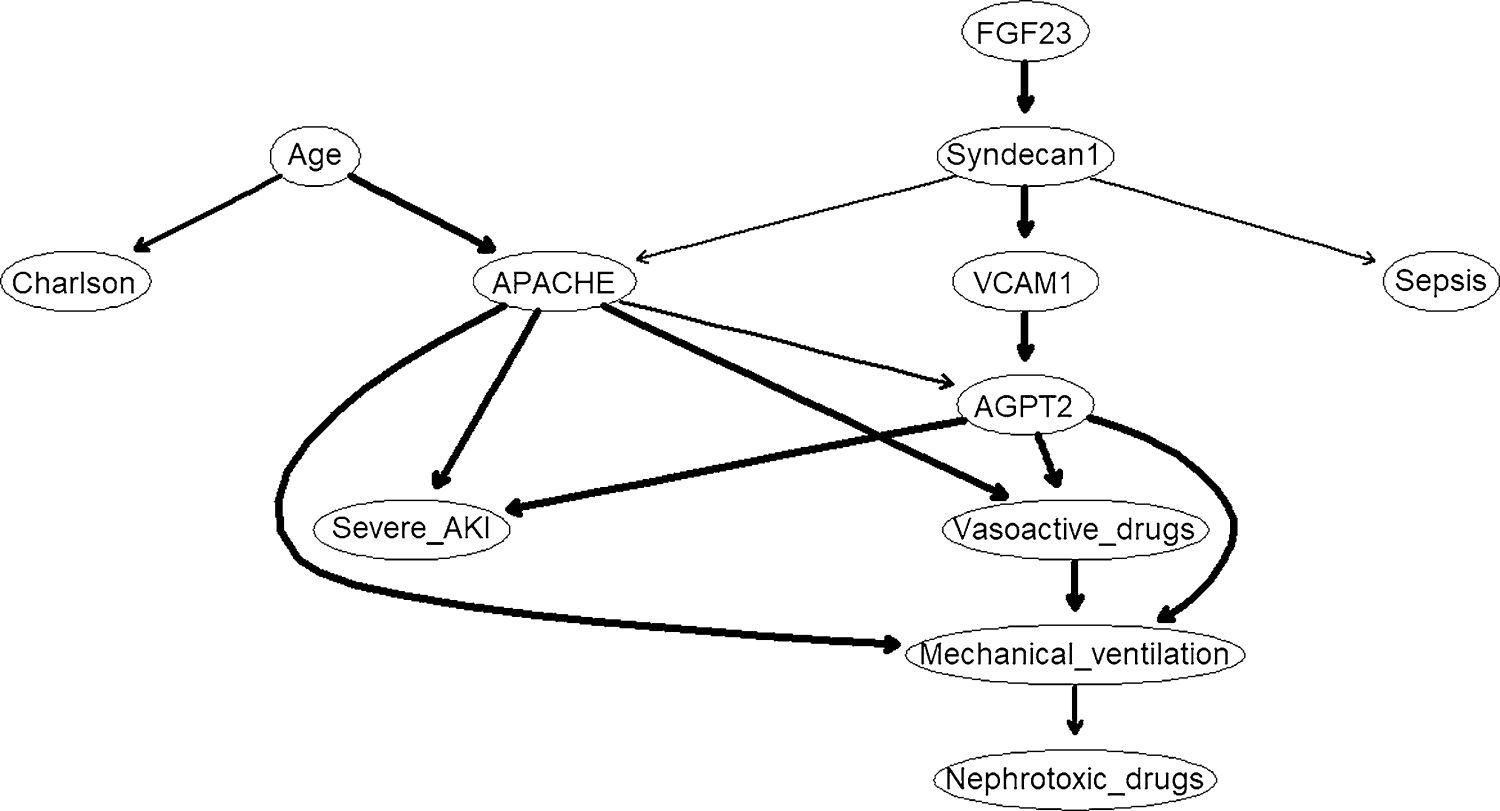
The DAG underlying the consensus Bayesian network learned from the variables measured on all patients. The thickness of the arcs is in the proportion to their strength; only arcs with a strength greater than 0.60 are included in the consensus network. Gender, body mass index, ICAM-1 and VEGF are not included in no pathway to severe AKI

To explore possible causal associations between FGF-23, VCAM-1, AGPT2 and syndecan-1, we performed Bayesian network learning—Fig. 4. We can observe FGF-23 is mediated by a pathway including syndecan-1 → VCAM-1 → AGPT2 → severe AKI. In order to validate the model learning strategy we perform 10 runs of 10-fold cross-validation and measure the predictive accuracy for Growth given all the other variables. The result is a classification error of ≈ 0.03.

## Discussion

Increased FGF23 is frequently seen in critically-ill patients, and has emerged in recent years as an established risk factor for acute kidney injury in this population [6, 7, 9]. Endothelial cells express FGF23 receptors and, although direct vascular effects of FGF23 remain largely elusive, it has been suggested that FGF23 directly impairs endothelial-dependent vasorelaxation [23]. In CKD patients, FGF23 is associated with impaired endothelial-dependent relaxation, endothelial-independent relaxation, arterial stiffness and cardiovascular disease [13]; however, the effects of FGF23 in the acute setting are largely unknown. In this cohort of critically-ill patients, we measured FGF23 and several endothelial-related biomarkers to test the hypothesis that the association of FGF23 and severe AKI could be mediated by endothelium/glycocalyx damage. We confirmed an independent association between FGF23 and severe AKI. Additionally, FGF23 was also associated with all, except VEGF, endothelial-related biomarkers. Moreover, the association between FGF23 and severe AKI was mediated by higher plasma levels of endothelial-related biomarkers—VCAM-1, syndecan-1 and AGPT2. In the DAG model, including the three endothelial-related biomarkers, the suggested pathway discloses endothelial-related biomarkers are mediators in the causal association between FGF23 and severe AKI. Our findings provide evidence supporting the hypothesis that an increased FGF23 leads to severe AKI through endothelium/glycocalyx lesion.

Whether FGF23 is simply a disease severity marker or directly contributes to adverse outcomes is a key question that could not be answered by either previous or present observational studies; but in addition to endothelial damage, other possible mechanisms explaining the acute adverse effects of FGF23 on renal function include: immune dysfunction [25], inflammation [26] and via inhibitory effects on vitamin D metabolite activation [31]. Although our studies and others have not been able to find an independent effect of vitamin D metabolites on AKI when evaluating FGF23 [7], it is possible that tissue-level paracrine effects of FGF23 on vitamin D activation and degradation can occur in the absence of major changes in circulating vitamin D levels, and even contribute to endothelial dysfunction [7, 32]. In summary, it is not possible to rule out the presence of other mechanisms related to FGF23, in addition to endothelial dysfunction.

The pathophysiology of endothelial damage is largely unknown, mainly that of its inner component, the glycocalyx. Although the most frequent suggested pathway point to syndecan-1 as the first mediator of FGF23 effect on severe AKI, it is not possible to conclude whether endothelial cell damage leads to glycocalyx dysfunction or if the opposite is true. In fact, the interrelationship between endothelial biomarkers are probably bidirectional pathways [33]. In the presented DAG, no clinical variable was directly associated with FGF23, explaining previous results disclosing independent association between FGF23 and AKI. Our results also confirm syndecan-1 is associated with sepsis as demonstrated in other studies [34, 35] and the strong association between AGPT4 and mechanical ventilation—Fig. 4 [36].

Because of its pleiotropic effects, as stated above, one possible candidate to the mediation of FGF 23 action on endothelium is vitamin D; however, we did not include it in the mediation analysis, as we found no significant association between vitamin D metabolites and severe AKI, ruling out the second principle to consider an element as a possible mediator (see “Statistical analysis” section). Because of economic constraints, we measured iPTH and vitamin D metabolites only in a subgroup of patients; however, other studies have demonstrated a lack of association between vitamin D and AKI [7, 8].

There are several limitations to our study. First, the most obvious limitation is the cross-sectional design and, therefore, it cannot show causality. Mediation analysis and Bayesian network learning provides evidence supportive of potential causal pathways that must then be confirmed by appropriate interventional studies in animal models or clinical trials. Although we adjusted the models for several known potential confounders, it is possible that the described effects may be attributable to unknown variables. Moreover, our proposed sequence of endothelial-related biomarkers mediators was only statistically-based and biological confirmation of this hypothesis is required. Second, although this is a prospective cohort, the great majority of patients did not have available information on sCr before hospital admission, Because our objective was to evaluate the effects of biomarkers on developing AKI (new or worsening), the first serum creatinine measurement available after ICU admission collected together with the biomarker samples was used as the reference value for renal function. Third, we did not measure intact FGF23; however, we consider other studies demonstrated that the association of C-terminal with AKI is similar to the intact assay [8]. Finally, although we adjusted for several potential confounders, our results may have been affected by residual confounding effects.

## Conclusions

Our study suggests the association between FGF23 and AKI are mediated by endothelial-related biomarkers, mainly VCAM-1, syndecan-1 and AGPT2. Moreover, the statistical models show that syndecan-1, a biomarker of the endothelial glycocalyx dysfunction, seems to be the first mediator between FGF23 and severe AKI.

## Additional files

**Additional file 1: Table S1.** FGF23 and endothelial-related biomarkers’ Spearman correlations.

**Additional file 2: Table S2.** Endothelial-related biomarkers stratified by AKI status.

**Additional file 3: Table S3.** Association of mineral biomarkers with severe AKI.

**Additional file 4: Figure S1.** Mediation analyses of the association between FGF23 and severe AKI in patients with diabetes mellitus.

## Abbreviations

AGPT2: angiopoietin 2
AKI: acute kidney injury
APACHE: Acute Physiology and Chronic Health Evaluation
CKD: chronic kidney disease
FGF23: fibroblast growth factor 23
ICAM-1: intercellular adhesion molecule-1
ICU: intensive care unit
iPTH: intact parathyroid hormone (PTH)
KDIGO: kidney disease improving global outcomes
VCAM-1: vascular cell adhesion protein 1
vitD: vitamin D
