# Supplementary material for: Fibroblast growth factor 23, endothelium biomarkers and acute kidney injury in critically-ill patients
Source: J Transl Med. 2019 Apr 11;17:121. doi: 10.1186/s12967-019-1875-6 (PMC6458699; doi:10.1186/s12967-019-1875-6)
Supplement: Supplementary file 1 — Additional file 1: Table S1. FGF23 and endothelial-related biomarkers’ Spearman correlations. [file 12967_2019_1875_MOESM1_ESM.doc]

| Biomarker | FGF23 | VCAM-1 | AGPT2 | Syndecan-1 | ICAM-1 |
| --- | --- | --- | --- | --- | --- |
| VCAM-1 | 0.365** | - | - | - | - |
| AGPT2 | 0.334** | 0.510** | - | - | - |
| Syndecan-1 | 0.412** | 0.444** | 0.357** | - | - |
| ICAM-1 | 0.297** | 0.561** | 0.530** | 0.329** | - |
| VEGF | -0.011 | 0.134* | 0.210* | 0.079 | 0.249** |

**Additional file 1:** **Table S1:** FGF23 and endothelial-related biomarkers’ Spearman correlations.

FGF23: fibroblast growth factor 23; VCAM-1: vascular cell adhesion protein 1; AGPT2: angiopoietin-2; ICAM-1: intercellular adhesion molecule-1; VEGF: vascular endothelial growth factor. *p<0.05 **p<0.001
