# Supplementary material for: Fibroblast growth factor 23, endothelium biomarkers and acute kidney injury in critically-ill patients
Source: J Transl Med. 2019 Apr 11;17:121. doi: 10.1186/s12967-019-1875-6 (PMC6458699; doi:10.1186/s12967-019-1875-6)
Supplement: Supplementary file 2 — Additional file 2: Table S2. Endothelial-related biomarkers stratified by AKI status. [file 12967_2019_1875_MOESM2_ESM.doc]

Additional file 2: **Table S2:** Endothelial-related biomarkers stratified by AKI status.

| **Biomarker** | **No-severe AKI (n=183)** | **Severe AKI (n=82)** | **p** |
| --- | --- | --- | --- |
| **VCAM-1, ng/mL** | 379 (247-586) | 667 (357-855) | <0.001 |
| **AGPT2, pg/mL** | 7,250 (3,180-17,050) | 14,845 (6,920-28,755) | <0.001 |
| **Syndecan-1** | 62.2 (43.3-118.1) | 142.7 (55.9-355.8) | <0.001 |
| **ICAM-1** | 644 (462-794) | 687 (501-998) | 0.020 |
| **VEGF** | 114.1 (69.9-201.1) | 136 (74-246) | 0.157 |
