# Supplementary material for: Fibroblast growth factor 23, endothelium biomarkers and acute kidney injury in critically-ill patients
Source: J Transl Med. 2019 Apr 11;17:121. doi: 10.1186/s12967-019-1875-6 (PMC6458699; doi:10.1186/s12967-019-1875-6)
Supplement: Supplementary file 3 — Additional file 3: Table S3. Association of mineral biomarkers with severe AKI. [file 12967_2019_1875_MOESM3_ESM.doc]

Additional file 3: **Table S3:** Association of mineral biomarkers with severe AKI.

|  | **Crude OR (95%CI)** | **Adjusted OR (95%CI)** |
| --- | --- | --- |
| **Serum Phosphorus** | 1.17 (0.64-2.18) | 1.02 (0.44-2.38) |
| **Serum Calcium** | 1.06 (0.72-1.85) | 0.94 (0.67-1.75) |
| **iPTH** * | 1.12(0.63-1.88) | 0.99 (0.46-2.12) |
| **25-vitamin D** * | 1.02(0.53-1.62) | 1.07(0.55-2.04) |
| **1,25-vitamin D** * | 0.82(0.32-1.48) | 0.91(0.36-2.22) |

* nested case-control subcohort (n=84)

Adjusted for age (per year), gender, main comorbidities, baseline estimated glomerular filtration rate, serum hemoglobin, need for vasoactive drugs, mechanical ventilation, sepsis and nephrotoxin exposure.
