# Supplementary material for: Fibroblast growth factor 23, endothelium biomarkers and acute kidney injury in critically-ill patients
Source: J Transl Med. 2019 Apr 11;17:121. doi: 10.1186/s12967-019-1875-6 (PMC6458699; doi:10.1186/s12967-019-1875-6)
Supplement: Supplementary file 4 — Additional file 4: Figure S1. Mediation analyses of the association between FGF23 and severe AKI in patients with diabetes mellitus. [file 12967_2019_1875_MOESM4_ESM.docx]

Additional file 4: **Figure S1:** Mediation analyses of the association between FGF23 and severe AKI in patients with diabetes mellitus. Path models and mediation analyses describe mediation of the association between FGF23 and severe AKI through endothelial-related biomarkers individually. Path effects are reported as Odds-ratio scale of natural log-transformed values of biomarkers. Models are adjusted for age, gender, and APACHE II score at ICU admission. Residual direct effects are labeled as path A in each model, and indirect effects are labeled as letters B and C.

**MODEL A MODEL B**

**AGPT2**

**-1**

**FGF-23**

**B_2_**

**C_2_**

**A_2_**

**Severe AKI**

**VCAM-1**

**-1**

**B_1_**

**C_1_**

**FGF-23**

**A_1_**

**Severe AKI**

| **Path** | **Effect** | **95% CI** |
| --- | --- | --- |
| A_1_ | 0.21 | (0.02 – 0.89) |
| B_1_C_1_ | 0.09 | (0.01 – 0.64) |

| **Path** | **Effect** | **95% CI** |
| --- | --- | --- |
| A_2_ | 0.23 | (-0.19 – 1.01) |
| B_2_C_2_ | 0.09 | (0.06 – 0.88) |

**MODEL C**

**Syndecan-1**

**-1**

**FGF-23**

**B_3_**

**C_3_**

**A_3_**

**Severe AKI**

| **Path** | **Effect** | **95% CI** |
| --- | --- | --- |
| A_3_ | 0.17 | (0.01 – 0.97) |
| B_3_C_3_ | 0.07 | (0.03 – 0.86) |
